# A GA microsatellite in the *Fli1 *promoter modulates gene expression and is associated with systemic lupus erythematosus patients without nephritis

**DOI:** 10.1186/ar3189

**Published:** 2010-11-18

**Authors:** Erin E Morris, May Y Amria, Emily Kistner-Griffin, John L Svenson, Diane L Kamen, Gary S Gilkeson, Tamara K Nowling

**Affiliations:** 1 Division of Rheumatology, Department of Medicine, Medical University of South Carolina, 96 Jonathon Lucas St., Charleston, SC 29425, USA; 2 Department of Biostatistics and Epidemiology, Medical University of South Carolina, 86 Jonathon Lucas St., Charleston, SC 29425, USA

## Abstract

**Introduction:**

The transcription factor Fli1 is implicated in the pathogenesis of systemic lupus erythematosus (SLE). Recently, a GA_n _polymorphic microsatellite was characterized in the mouse *Fli1 *promoter that modulates promoter activity and is truncated in two lupus mouse models compared to non-autoimmune prone mice. In this work, we characterize a homologous GA_n _microsatellite in the human *Fli1 *promoter. The purpose of this study is to determine the effect of the microsatellite length on *Fli1 *promoter activity *in vitro *and to determine if the length of the GA_n _microsatellite is associated with SLE and/or specific disease characteristics.

**Methods:**

Constructs with variable lengths of the GA_n _microsatellite in the *Fli1 *promoter were generated and analyzed in promoter/reporter (P/R) assays in a human T cell line. Using three SLE patient cohorts and matched controls, microsatellite length was measured and association with the presence of disease and the occurrence of specific disease manifestations was assessed.

**Results:**

P/R assays demonstrated that the presence of a shorter microsatellite resulted in higher *Fli1 *promoter activity. A significant association was observed in the lupus cohort SLE in Gullah Health (SLEIGH) between the GA_26 _base pair allele and absence of nephritis.

**Conclusions:**

This study demonstrates that a GA_n _microsatellite in the human Fli1 promoter is highly polymorphic. The length of the microsatellite is inversely correlated to *Fli1 *promoter activity in a human T cell line. Although no association between microsatellite length and lupus was observed, an association between a specific microsatellite length and patients without nephritis in the SLEIGH cohort was observed.

## Introduction

Systemic lupus erythematosus (SLE) is a prototypic autoimmune disease characterized by the production of autoantibodies, formation of immune complexes and subsequent deposition in target tissues with resultant local inflammation and organ damage [1]. Nearly every organ system can be involved in lupus with the most prominent being the kidneys, joints, skin and brain [1]. The major determinant of morbidity and mortality is renal involvement, although infection and cardiovascular disease are leading causes of death. The American College of Rheumatology outlines the most common disease outcomes of SLE in the 1997 revised classification criteria including arthritis, serositis, nephritis, immune-mediated cytopenias, and lupus-specific autoantibody positivity. Commonly, the course of disease will include periods of remission and flares and the disease presentation is heterogeneous among patients making SLE a difficult disease to characterize, diagnose, and study. Similar to most autoimmune diseases, lupus is believed to result from an environmental event triggering disease in a genetically susceptible individual.

Increasing evidence suggests that dysregulation of the transcription factor Fli1 contributes to the pathogenesis of lupus. Fli1 is a member of the Ets family of transcription factors and is preferentially expressed in endothelial and hematopoietic cell lineages. Levels of expression are linked to the pathogenesis of lupus. During lymphoid development, Fli1 is highly expressed in immune cells including mature B cells, pre-T cells, and resting, mature T cells [2]. Over-expression of Fli1 in peripheral blood mononuclear cells (PBMCs) in lupus patients is correlated with disease activity [3]. Fli1 is over-expressed in several lupus mouse models including T cells of NZB/NZW f1 mice and spleen of MRL/lpr mice [3,4]. Transgenic global over-expression of Fli1 in healthy, non-autoimmune prone mice results in a lupus-like phenotype with the presence of autoreactive lymphocytes, autoantibodies and the development of immune complex mediated kidney disease [5]. Conversely, reduction of Fli1 levels by 50% in MRL/lpr and NZM2410 lupus-prone mice improved the disease phenotypes in these models by decreasing autoantibody production and kidney disease and significantly prolonging survival [4,6]. This evidence demonstrates that expression levels of Fli1 in lupus affect disease phenotype.

We demonstrated previously that a polymorphic microsatellite consisting of GA repeats within the proximal promoter of the mouse *Fli1 *gene is shorter in the MRL/lpr and NZM2410 lupus mouse models compared to non-autoimmune prone BALB/c and C57BL/6 mice [7]. Promoter/reporter (P/R) assays demonstrated that *in vitro *activity of the mouse *Fli1 *promoter in a T cell line was inversely correlated with microsatellite length. The murine and human *Fli1 *promoters are highly homologous with 88% homology in the proximal promoter region, including the GA microsatellite [8]. Together, these studies suggested that a shorter microsatellite in the Fli1 promoter may contribute to over-expression of Fli1 and to the pathogenesis of lupus. Using clinical data and genomic DNA specimens from SLE patients and controls, we demonstrate that the human microsatellite shows a similar inverse correlation between length and promoter activity in a human T cell line and that a microsatellite length of GA_26 _is significantly more prevalent in SLE patients without nephritis and tended to be more prevalent in SLE patients with serositis.

## Materials and methods

### Plasmid constructs

The promoter/reporter (P/R) constructs containing the -502 to -37 region of the human *Fli1 *gene were generated from genomic DNA isolated from control subjects in the Carolina Lupus Study as described previously [8]. The -502/-37 P/R constructs analyzed were identical except for the difference in microsatellite lengths; 28, 24, 16 or 13 GA repeats. Two clones for each of the microsatellite lengths were generated and tested. All constructs were confirmed by direct sequencing.

### Transfections

pGL3 h*Fli1*-502/-37 P/R constructs were transfected into the Jurkat human T cell line using Fugene (Pierce, Rockford, IL USA) following the manufacturer's directions. A Renilla luciferase construct was co-transfected to normalize for transfection efficiency. Cells were harvested 24 hours after transfection and cell lysates were analyzed for luciferase expression using the dual luciferase detection kit (Promega, Madison, WI USA) and quantified using a luminometer. Promoter activity as a measure of luciferase expression was compared to the pGL3 Basic empty vector. Transfections were performed at least three times in duplicate with two different clones and averaged.

### Cells

The Jurkat human T cell line was maintained in RPMI 1640 with 10% fetal bovine serum and antibiotics at 37°C and 5% humidity. Cells were passaged the day prior to transfection.

### Microsatellite fragment length measurements

The microsatellite containing region of the *Fli1 *proximal promoter was amplified from genomic DNA from subjects in the Carolina Lupus study (CLU), SLE in Gullah Health study (SLEIGH) and the MUSC Lupus Clinic study (Clinic) using the following primers: upstream primer, hGA2Up, 5'-/56-FAM/ATGTGTCTGGGCATCTC-3', contains a FAM fluorescent tag; and downstream primer, GADn, 5'-GCTAATTTTGGGAAGTGACT-3'. The amplified, FAM-tagged PCR products were sent to the DNA Facility at Iowa State University (Ames, IA, USA) for high throughput genotyping analysis using the Applied Biosystems 3100 Genetic Analyzer (Carlsbad, CA, USA) and size marker. Several samples, including those that were used to generate the P/R constructs, were run across multiple plates to ensure consistent amplification and sizing across plates and over half of the samples were run twice. The raw sizing data were visually analyzed for peak quality followed by analysis and binning using the GeneMapper software (Applied Biosystems, Carlsbad, CA, USA). Direct sequencing of 32 of the amplified products of various lengths demonstrated that the differences in amplified product sizes were due to the length of the microsatellite and not to other sequence changes.

### Subjects

This study utilized subject data from three lupus cohorts. Genomic DNA was isolated from peripheral blood samples obtained from the study cohorts taken upon receiving informed consent and in compliance with the Institutional Review Board for Human Studies. All aspects of this study were conducted according to the Helsinki Declaration. The Carolina Lupus (CLU) cohort is a case-control study investigating genetic and environmental factors predisposing individuals to SLE [9]. Patients enrolled in the CLU study were recruited through university and community rheumatology in eastern North Carolina and South Carolina. Patients met at least 4 of the 11 revised American College of Rheumatology classification criteria for SLE [10,11]. All patients were diagnosed with SLE between 1 January 1995 and 1 July 1999 and were enrolled in the CLU study within one year of diagnosis. Matched control subjects were recruited from state driver's license registries. At the time of enrollment, blood samples were taken from the study subjects for the extraction of genomic DNA.

The Systemic Lupus Erythematosus in Gullah Health (SLEIGH) study includes African American lupus patients and controls living on the Sea Islands of the South Carolina and Georgia coasts [12]. SLEIGH subjects represent a unique genetic group with a low percent admixture of non-African genes. Subjects enrolled in the SLEIGH study were self-identified as a member of the African American Gullah community with no known ancestors that were not of Gullah lineage. Patients in SLEIGH met at least 4 of the 11 American College of Rheumatology classification criteria for SLE [10,11]. Patients were identified as multiplex if the diagnosis of SLE could be documented in one or more family members. Population controls in SLEIGH used for our analyses had no known family history of SLE or other autoimmune disease and were matched on age and gender to patient cases. Blood samples were taken from the study subjects at the time of enrollment for extraction of genomic DNA.

Caucasian and African American lupus patients from the MUSC clinic that met 4 of the 11 American College of Rheumatology classification criteria for SLE were included for data collection. Blood samples were taken for extraction of genomic DNA.

The clinic patients were included with the CLU study for analyses. Separate analyses were performed on the Caucasian and African American populations in order to avoid possible confounders due to population stratification. Initial analyses for the SLEIGH study data considered only patients not from multiplex families. Additional analyses were performed on the SLEIGH study data to include one multiplex patient chosen at random from each multiplex family. Within each cohort, three statistical testing approaches were considered.

### Statistics

P/R assay data were analyzed by the Student's *t*-test to identify statistically significant differences. To begin analyses of the patient cohort data, hypothesis-generating genotype tests were considered by dichotomizing into short/long alleles and using 2-df Fisher's exact tests (or chi-squared tests where the counts of genotypes were above five for each possible genotype) to test for association with disease between cases and controls or individual disease characteristics within cases. Next, average allele length was compared between cases and controls using a two-sample *t*-test. Lastly, due to the large number of alleles observed, the CLUMP program was used to compare each allele to all the other possible alleles. The CLUMP program, described by Sham and Curtis [13], allows testing each allele frequency against all other allele frequencies, using a chi-squared test statistic. Statistical analyses were performed separately on patients from the SLEIGH study while patients in the CLU and MUSC clinic cohorts were analyzed together.

## Results

### A GA_n _dinucleotide repeat in the proximal promoter of the human Fli1 gene modulates *in vitro* promoter activity in T cells

**Figure 1:**
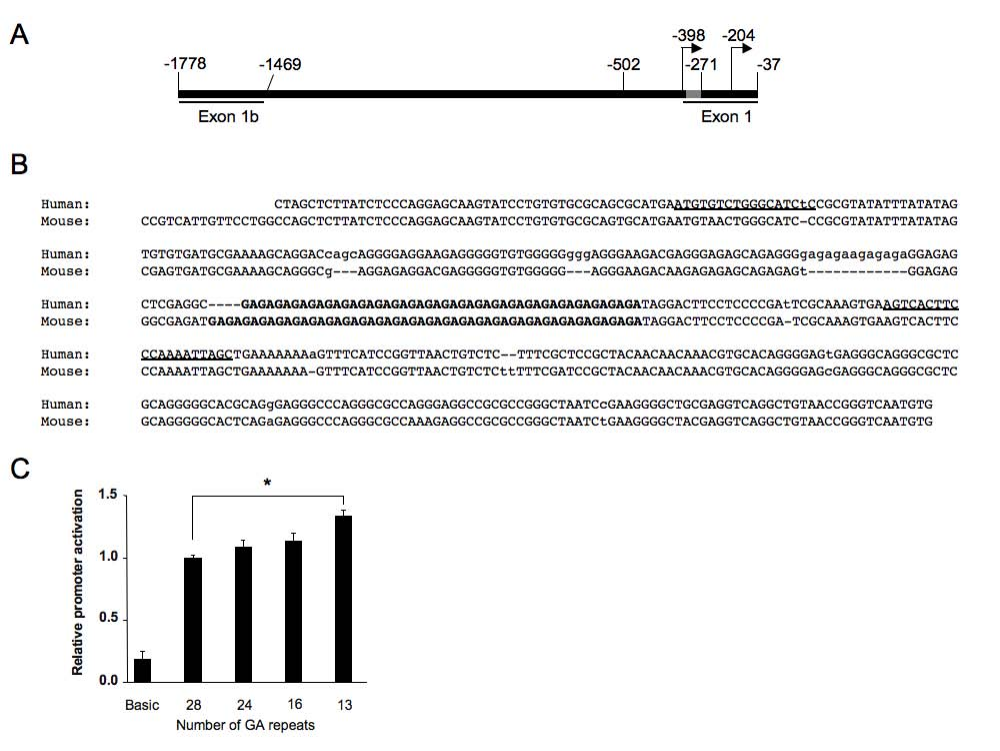
**A GA_n _microsatellite in the hFli1 promoter modulates activity in a human T cell line**. **A**. Construct showing the location of distal and proximal promoters, transcription start sites (arrows) and GA microsatellite (white box) in the Fli1 promoter. Numbering is relative to the +1 translation start site. **B**. Sequence of the human -502/-37 region is aligned with the equivalent -505/-37 region from mouse. The GA microsatellite is in bold and primer sites used to amplify the microsatellite-containing region in human genomic DNA samples are underlined. **C**. The -502 to -37 region of the human Fli1 promoter shown in B with different lengths of the GA_n _microsatellite was cloned from genomic DNA samples of unaffected controls in the CLU study into the pGL3 Basic reporter vector. Constructs were transfected into Jurkat T cells and assayed for promoter activity. Expression is presented relative to the pGL3 Basic empty vector, which was set to 1. Results are an average of three independent transfections performed with two independently derived clones. *P < 0.005.

A polymorphic GA_n _dinucleotide microsatellite is present in the proximal region of the human *Fli1 *promoter 271 base pairs upstream (-271) from the start site of translation (Figure 1A, B). The location of this GA_n _repeat is homologous to the polymorphic microsatellite we recently analyzed in the mouse *Fli1 *promoter [7]. In our previous study, deletion analyses of the human *Fli1 *promoter demonstrated that the microsatellite is not required for full promoter activity in a T cell line [8]. However, the microsatellite was demonstrated to modulate the activity of the mouse promoter in T cells such that the shorter the microsatellite the greater the promoter activity [7].

To determine if there is a similar inverse correlation between microsatellite length and promoter activity with the human *Fli1 *promoter, P/R constructs containing microsatellites of 28, 24, 16, and 13 GA repeats were transfected into the Jurkat human T cell line. The pGL3 construct containing 13 GA (GA_13_) repeats resulted in the highest level of *Fli1 *promoter activity (Figure 1C). Promoter activity decreased as microsatellite length increased with a statistically significant difference between the longest (GA_28_) and the shortest (GA_13_) alleles (*P *< 0.001). This demonstrates that relative *Fli1 *promoter activity is inversely correlated with the number of GA repeats in this human T cell line similar to our previous study of the mouse *Fli1 *promoter [7].

### Distribution of microsatellite length in patients and control subjects

In a previous study, expression of the Fli1 gene was shown to be elevated in T cells from SLE patients compared to unaffected control subjects [3]. Based on our results demonstrating that the length of the microsatellite is inversely correlated to Fli1 promoter activity and that a shorter microsatellite is present in lupus-prone mouse strains [7], we sought to determine whether the length of the microsatellite is associated with SLE. The microsatellite-containing region of the *Fli1 *promoter was amplified and length measured in the Carolina Lupus (CLU) study cohort, MUSC Lupus Clinic (Clinic) study cohort and the SLEIGH study cohort. Demographics are presented in Table 1. The CLU and Clinic cohorts were analyzed together and include 197 SLE patients and 162 unaffected controls. The SLEIGH cohort includes 154 patients and 97 unaffected controls and for statistical analyses, the cohort data was divided into two groups that either excluded multiplex families or included one randomly selected patient from each multiplex family.

**Table 1:** Demographics of the study cohorts

	Controls		Patients	
		
	Total Numbers	Age Range (Median Age)	Total Numbers	Age Range (Median Age)
CLU/MUSC Clinic Caucasians	110	18-75 (39)	62	18-51 (36)
CLU/MUSC Clinic African Americans	52	16-61 (33)	135	15-54 (37)
SLEIGH Without multiplex patients	97	11-74 (42)	123	10-69 (37)
SLEIGH With multiplex patients	97	11-74 (42)	154	10-70 (39)

The number of patients and controls and the median age ranges for the Carolina Lupus (CLU) study, Medical University of South Carolina (MUSC) clinic, and System Lupus Erythematosus in Gullah Health (SLEIGH) study participants.

**Figure 2:**
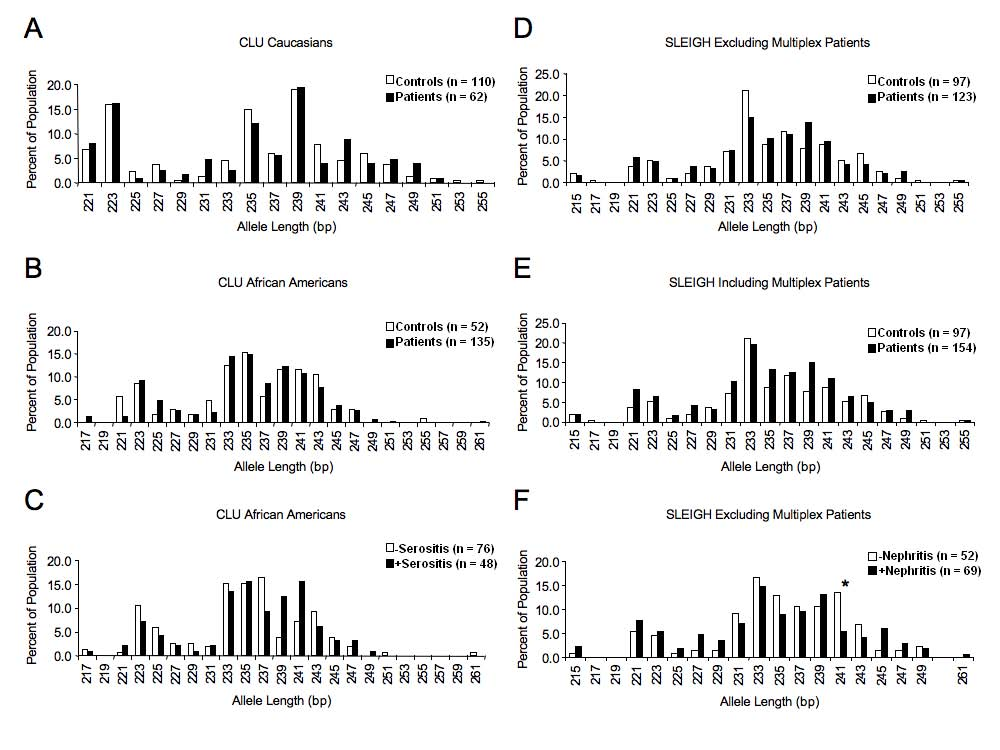
**Distribution of the GA_n _microsatellite allele in patient and control subjects of the CLU and SLEIGH cohorts**. **A**. CLU Caucasian subjects. **B**. CLU African American subjects. **C**. African American patient population of the CLU cohort divided with respect to serositis occurrence. **D**. SLEIGH subjects excluding multiplex patients (not shown on graph is one patient with an allele of 267 base pairs). **E**. SLEIGH subjects including multiplex patients (not shown on graph is one patient with an allele of 267 base pairs). **F**. SLEIGH patient population divided with respect to nephritis occurrence. *P < 0.05. All n values are representative of the number of patients, each patient having two alleles.

Interestingly, following genotyping of these cohorts we observed over 20 different alleles with a range of GA repeats from 13 to 39 within these populations, indicating that this microsatellite is highly polymorphic in humans. Allele distributions for each cohort are presented in Figure 2. Due to the large number of alleles, the subjects were grouped into short and long alleles for statistical analyses to determine if a shorter GA microsatellite is associated with disease. Next, we compared average microsatellite length between cases and controls. No association was observed with either of these analyses (data not shown).

We performed additional analyses to explore other phenotype-genotype associations that were not initially hypothesized, specifically the possibility that a particular length may be associated with disease or a disease phenotype. Analyses of length in cases and controls in the CLU cohort were separated into Caucasians (Figure 2A) and African Americans due to concerns of population stratification (Figure 2B). In general, the distribution of alleles was similar in cases and controls. These results indicate no significant differences in the overall distribution of microsatellite length between SLE patients and unaffected controls in the CLU cohort regardless of ethnicity. The same analyses were performed with the SLEIGH cohort data. As in the CLU cohort, the overall distribution was similar between cases and controls whether the multiplex patients were excluded (Figure 2D) or included (Figure 2E). These results demonstrate no significant differences in the overall distribution of microsatellite length between SLE patients and unaffected controls in the SLEIGH cohort regardless of inclusion of multiplex patients.

### Assessment of microsatellite length and lupus disease characteristics

To determine whether microsatellite length is associated with specific disease characteristics, microsatellite length was analyzed within the patient population of the CLU and SLEIGH cohorts. Analyses conducted include microsatellite length and occurrence of arthritis, serositis, nephritis, immune-mediated cytopenias, and lupus-specific autoantibody positivity. These disease characteristics were defined using the 1997 revised American College of Rheumatology SLE classification criteria [11]. The disease characteristic could have occurred at any point in the patient's history, however, must be attributable to lupus. As with allele distribution analysis, no significant associations were observed in analyses of short and long alleles or average allele length with disease phenotypes (data not shown).

We then analyzed the occurrence of specific allele lengths with disease phenotypes. No significant associations were observed between microsatellite length and arthritis, cytopenias, or lupus-specific autoantibody positivity in the CLU Caucasian, CLU African American or SLEIGH populations (data not shown). Analyses of allele length with nephritis in the SLEIGH cohort, excluding multiplex patients, identified the 241 bp allele (GA_26_) as being more prevalent in patients that did not develop nephritis (Figure 2F). This difference was statistically significant after adjusting for multiple comparisons (*P *< 0.05) (Table 2). These results suggest that the 241 base pair allele may be protective against the development of nephritis in SLE patients in the SLEIGH population.

**Table 2:** Summary of results

Alleles associated with patients	CLU		SLEIGH	
		
	Caucasian (*n *= 62)	AA (*n *= 135)	Without Multiplex (*n *= 123)	With Multiplex (*n *= 154)
With Serositis	none	239/241	239	239
Without Nephritis	none	none	241 (p < 0.05)	241

Summary of results for the Carolina Lupus (CLU) study African American (AA) and Caucasian participants and for the Systemic Lupus Erythematosus in Gullah Health (SLEIGH) participants.

The 239 bp (GA_25_) and 241 bp (GA_26_) alleles were more prevalent in patients with serositis compared to patients without serositis in the African American CLU patients (Figure 2C and Table 2). Similarly, as shown in the results summary in Table 2 the 239 bp (GA_25_) allele was more prevalent in patients with serositis in the SLEIGH cohort. However, these differences did not remain statistically significant after adjusting for multiple comparisons. We then combined the SLEIGH cohort, which is entirely African American, and the CLU African American populations to determine whether increasing our sample size would result in significant differences. Although the 239 bp (GA_25_) allele length remained more prevalent when combining the two populations, statistical significance was not obtained (data not shown).

## Discussion

We previously demonstrated that a polymorphic GA_n _microsatellite in the mouse *Fli1 *promoter modulates promoter activity in a T cell line and is evolutionarily conserved [7]. Although the GA_n _microsatellite in the mouse and human promoters is not required for promoter activity *in vitro *[7,8], varying the length of the microsatellite in the mouse promoter modulated promoter activity in T cells with the length of the microsatellite being inversely proportional to promoter activity [7]. Here we determined that the *in vitro *activity of the human *Fli1 *promoter also decreased as the number of GA repeats in the microsatellite increased (Figure 1C). The inverse relationship between microsatellite length and *Fli1 *promoter activity observed in mouse and human suggests a possible functional role of the GA_n _microsatellite in the regulation of Fli1 expression.

Microsatellites occur at high frequency in the protein coding and non-coding regions of the human genome, which has raised many questions about their function in normal development and disease states [14]. Although there are many examples in the literature of the ability of microsatellites to affect expression of genes (including EGFR, estrogen receptor β, Kv1.5, nucleolin, acetyl CoA carboxylase, heme oxygenase I, matrix metalloproteinase 9, Cyr61, heat shock genes, collagen Iα2, and Pax6 [15-26]), the mechanisms involved are not entirely understood. Microsatellites are thought to function primarily by altering chromatin structure. Specifically, properties of GA microsatellites include the ability to adopt H-form DNA and bind GAGA factor. The H-DNA conformation includes both single- and triple-stranded regions that are DNase I-hypersensitive [27] and may provide an open chromatin configuration that allows binding of transcription factors to neighboring regulatory elements [28,29]. GAGA factor is a protein identified in Drosophila and its binding to GA-rich sequences in promoters can repress or activate transcription through effects on nucleosomes [30-33]. Recently, the vertebrate homolog of GAGA factor, th-POK, was identified and demonstrated to bind GA-rich sequences [34]. Additionally, GA-rich sequences are thought to play a role as enhancer-blocking or insulator elements [35]. The Fli1 GA microsatellite was demonstrated previously to be sensitive to nuclease S1 digestion and shown to form H-DNA *in vitro *[36,37]. Our *in vitro *experiments with both the mouse and human promoters support a role of the microsatellite in dampening promoter activity as length increases but not in complete repression of activity. How this microsatellite functions *in vivo *remains to be determined.

Previous results demonstrated that Fli1 expression is increased in mouse models of lupus [3,4] and in T cells of SLE patients [3] compared to unaffected controls. Interestingly, globally decreasing Fli1 levels by 50% in two different lupus mouse models resulted in significantly improved disease, most notably improved nephritis, accompanied by significantly prolonged survival [4,6]. Furthermore, it was demonstrated that reducing Fli1 levels by 50% in hematopoietic cells in a lupus mouse model also resulted in improved disease and survival [38]. Together these studies demonstrated that modulating Fli1 levels plays an important role in the progression of lupus. Based on these observations and our P/R results demonstrating that the length of the microsatellite modulates Fli1 expression, we hypothesized that a shorter microsatellite in the Fli1 promoter may lead to over-expression of Fli1 and thus may be associated with lupus or with specific disease phenotypes such as nephritis. The association of microsatellites with the occurrence of several diseases including SLE, rheumatoid arthritis, lung fibrosis, epithelial ovarian cancer, thyroid cancer, acute pancreatitis, breast cancer, and multiple neurological diseases has been reported [39-48].

We expected the microsatellite to be polymorphic in the human population; however, we were surprised to observe more than 20 different alleles spanning 13 to 39 GA repeats in the populations tested, including the highly genetically related SLEIGH population. The highly polymorphic nature of this microsatellite is interesting and prompted multiple analyses to explore all possible phenotype-genotype associations. Although the GA_n _microsatellite length had an effect on *Fli1 *promoter activity *in vitro*, no association was identified between microsatellite allele length and the occurrence of SLE in the SLEIGH and CLU cohorts. The pathogenesis of SLE is complex and it is likely that the disease results from alterations in the expression of multiple target genes. Thus, Fli1 may contribute to the progression of disease but we believe it is more likely to be associated with a specific disease characteristic.

To determine if GA microsatellite length is associated with specific aspects of SLE we compared the presence of lupus-specific characteristics with microsatellite length. No association was observed between microsatellite length and arthritis, cytopenia or lupus specific autoantibodies. To our knowledge, it is unknown whether Fli1 plays a role in the development of arthritis or cytopenia in lupus, although it was demonstrated that reducing Fli1 levels in a lupus mouse model had no effect on total B cell or T cell numbers [4]. Therefore, it is not unexpected that no association between the Fli1 microsatellite and arthritis or cytopenia was observed. Based on results in lupus mouse models in which reducing Fli1 levels resulted in decreased autoantibody levels [4,6], we expected to observe an association of the Fli1 microsatellite with autoantibody production. However, the effect of Fli1 on autoantibody levels appears to be secondary to effects on B cell activation [4], which may account for the apparent lack of association of the Fli1 microsatellite with autoantibodies in our study.

The 241 bp allele (GA_26_), was significantly associated with SLE patients that did not have nephritis in the SLEIGH cohort (Figure 2F and Table 2). Alleles of 239 and 241 bp, 25 and 26 GA repeats respectively, were more prevalent, although not significantly, in African American CLU patients with serositis (Figure 2C and Table 2). *In vitro *constructs in this long size range (23 to 28 repeats) exhibited weaker activation of the *Fli1 *promoter in P/R assays (Figure 1C), suggesting that lower Fli1 expression may be protective against nephritis and contribute to serositis. In lupus mouse models in which Fli1 levels were reduced globally or specifically in hematopoietic cells, nephritis was improved [4,6,38]. Conversely, expression of Fli1 in mouse endothelial cells controls vascular maturation and is required to maintain vascular integrity [49]. We speculate that a reduction in *Fli1 *promoter activity and, hence, expression may disrupt endothelial cell function in lupus patients and contribute to exaggerated serositis.

These findings support previous hypotheses that the effects of Fli1 expression in different cell types are variable and aberrant expression of Fli1 can contribute to the development of disease. For example, in scleroderma, reduced expression of Fli1 in skin fibroblasts and endothelial cells is implicated in the fibrotic and vascular components of the phenotype [49], while in lupus elevated expression of Fli1 in mononuclear cells is thought to contribute to the disease phenotype [3]. It is conceivable that the 241 bp allele representing a microsatellite of 26 GA repeats may delineate a threshold length of the microsatellite. Individuals with a Fli1 GA_n _microsatellite at or above the threshold may have lower levels of Fli1 expression in their lymphocytes, which would be protective against developing lupus nephritis while individuals with a Fli1 GA_n _microsatellite at or above the threshold may have lower expression levels of Fli1 in their endothelial cells, which may contribute to serositis. However, we tested all dichotomies of short/long alleles and didn't observe significant differences, suggesting that increased and decreased expression of Fli1 may be modulated by a specific length of the GA microsatellite.

## Conclusions

In this work, we characterized a highly polymorphic microsatellite of GA repeats in the human *Fli1 *promoter. Variable lengths of the GA_n _microsatellite modulated *Fli1 *promoter activity *in vitro *in a human T cell line such that the shorter the microsatellite the greater the promoter activity. Although aberrant expression of *Fli1 *in PBMCs was observed previously in SLE patients [3], specific association between microsatellite length and SLE was not observed in this study. However, the GA_26 _microsatellite length was specifically associated with patients that did not have nephritis and tended to be more prevalent in African American patients with serositis. Due to the highly polymorphic nature of this microsatellite, a greater number of additional subjects in these cohorts would be required to increase the statistical power in order to determine significant associations for each allele and/or genotype, especially within the shortest and longest alleles, which appear to be relatively rare. The expression of Fli1 in different cell types may mediate pathological effects that contribute to the multifaceted role of Fli1 in SLE. A more precise understanding of how this microsatellite functions to modulate Fli1 expression in different cell types would be beneficial in determining whether this microsatellite may serve as therapeutic marker in lupus. Future studies are aimed at determining whether the length of the Fli1 GA_n _microsatellite correlates with Fli1 expression levels in primary human cells and how the GA microsatellite precisely functions to modulate expression.

## Abbreviations

CLU: Carolina Lupus Study; CTCF: CCCTC binding factor; PBMCs: peripheral blood mononuclear cell; P/R: promoter/reporter; SLE: Systemic Lupus Erythematosus; SLEIGH: Systemic Lupus Erythematosus in Gullah Health.

## Competing interests

The authors declare that they have no competing interests.

## Authors' contributions

EEM drafted the manuscript and contributed to organizing and analyzing data. MYA performed the real-time PCR experimentation, P/R transfections and data collection. EKG performed the statistical analyses of the data. JLS participated in the data analyses and writing of the manuscript. DLK and GSG provided the gDNA samples and demographic information for the cohorts and contributed to the data analyses. TKN conceived of the study, designed the experiments and contributed to all aspects of the data collection and analyses and drafting and editing of the manuscript. All authors read and approved of the final manuscript.

## References

[B1] Oates JC Gilkeson GS Mediators of injury in lupus nephritis Curr Opin Rheumatol 2002 14 498 503 10.1097/00002281-200209000-00003 12192244

[B2] Anderson MK Hernandez-Hoyos G Diamond RA Rothenberg EV Precise developmental regulation of Ets family transcription factors during specification and commitment to the T cell lineage Development 1999 126 3131 3148 10375504 10.1242/dev.126.14.3131

[B3] Georgiou P Markoulakou IG Green JE Dantis P Romano-Spica V Kottardid S Lautenberger JA Watson DK Papas TS Fischinger PJ Bhat NK Expression of ets family of genes in systemic lupus erythematosus and Sjogren's syndrome International Journal of Oncology 1996 9 9 18 21541474

[B4] Zhang XK Gallant S Molano I Moussa OM Ruiz P Spyropoulos DD Watson DK Gilkeson G Decreased expression of the Ets family transcription factor Fli-1 markedly prolongs survival and significantly reduces renal disease in MRL/lpr mice J Immunol 2004 173 6481 6489 15528390 10.4049/jimmunol.173.10.6481

[B5] Zhang L Eddy A Teng YT Fritzler M Kluppel M Melet F Bernstein A An immunological renal disease in transgenic mice that overexpress Fli-1, a member of the ets family of transcription factor genes Molecular and Cellular Biology 1995 15 6961 6970 8524263 10.1128/mcb.15.12.6961 PMC230951

[B6] Mathenia J R-C E Williams S Molano I Ruiz P Watson D Gilkeson G Zhang X Impact of Fli1-1 transcription factor on autoantibody and lupus nephritis in NZM2410 mice Clin Exp Immunol 2010 162 362 367 10.1111/j.1365-2249.2010.04245.x 20731671 PMC2996603

[B7] Nowling TK Fulton JD Chike-Harris K Gilkeson GS Ets factors and a newly identified polymorphism regulate Fli1 promoter activity in lymphocytes Molecular immunology 2008 45 1 12 10.1016/j.molimm.2007.05.018 17606295 PMC2045641

[B8] Svenson JL Chike-Harris K Amria MY Nowling TK The mouse and human Fli1 genes are similarly regulated by Ets factors in T cells Genes Immun 2010 11 161 172 10.1038/gene.2009.73 19829305 PMC2832078

[B9] Cooper GS Parks CG Treadwell EL St Clair EW Gilkeson GS Dooley MA Occupational risk factors for the development of systemic lupus erythematosus J Rheumatol 2004 31 1928 1933 15468355

[B10] Tan EM Cohen AS Fries JF Masi AT McShane DJ Rothfield NF Schaller JG Talal N Winchester RJ The 1982 revised criteria for the classification of systemic lupus erythematosus Arthritis Rheum 1982 25 1271 1277 10.1002/art.1780251101 7138600

[B11] Hochberg MC Updating the American College of Rheumatology revised criteria for the classification of systemic lupus erythematosus Arthritis Rheum 1997 40 1725 10.1002/art.1780400928 9324032

[B12] Kamen DL Barron M Parker TM Shaftman SR Bruner GR Aberle T James JA Scofield RH Harley JB Gilkeson GS Autoantibody prevalence and lupus characteristics in a unique African American population Arthritis Rheum 2008 58 1237 1247 10.1002/art.23416 18438839

[B13] Sham PC Curtis D Monte Carlo tests for associations between disease and alleles at highly polymorphic loci Ann Hum Genet 1995 59 97 105 10.1111/j.1469-1809.1995.tb01608.x 7762987

[B14] Buschiazzo E Gemmell NJ The rise, fall and renaissance of microsatellites in eukaryotic genomes Bioessays 2006 28 1040 1050 10.1002/bies.20470 16998838

[B15] Gebhardt F Burger H Brandt B Modulation of EGFR gene transcription by a polymorphic repetitive sequence--a link between genetics and epigenetics Int J Biol Markers 2000 15 105 110 10763151 10.1177/172460080001500120

[B16] Ugai K Nishimura K Fukino K Nakamura T Ueno K Functional analysis of transcriptional activity of cytosine and adenine (CA) repeats polymorphism in the estrogen receptor beta gene J Toxicol Sci 2008 33 237 240 10.2131/jts.33.237 18544915

[B17] Mori Y Folco E Koren G GH3 cell-specific expression of Kv1.5 gene. Regulation by a silencer containing a dinucleotide repetitive element J Biol Chem 1995 270 27788 27796 10.1074/jbc.270.52.30862 7499248

[B18] Rothenburg S Koch-Nolte F Rich A Haag F A polymorphic dinucleotide repeat in the rat nucleolin gene forms Z-DNA and inhibits promoter activity Proc Natl Acad Sci USA 2001 98 8985 8990 10.1073/pnas.121176998 11447254 PMC55360

[B19] Tae HJ Luo X Kim KH Roles of CCAAT/enhancer-binding protein and its binding site on repression and derepression of acetyl-CoA carboxylase gene J Biol Chem 1994 269 10475 10484 7908293

[B20] Hirai H Kubo H Yamaya M Nakayama K Numasaki M Kobayashi S Suzuki S Shibahara S Sasaki H Microsatellite polymorphism in heme oxygenase-1 gene promoter is associated with susceptibility to oxidant-induced apoptosis in lymphoblastoid cell lines Blood 2003 102 1619 1621 10.1182/blood-2002-12-3733 12730098

[B21] Shimajiri S Arima N Tanimoto A Murata Y Hamada T Wang KY Sasaguri Y Shortened microsatellite d(CA)21 sequence down-regulates promoter activity of matrix metalloproteinase 9 gene FEBS Lett 1999 455 70 74 10.1016/S0014-5793(99)00863-7 10428474

[B22] Wang B Ren J Ooi LL Chong SS Lee CG Dinucleotide repeats negatively modulate the promoter activity of Cyr61 and is unstable in hepatocellular carcinoma patients Oncogene 2005 24 3999 4008 10.1038/sj.onc.1208550 15782120

[B23] Sandaltzopoulos R Mitchelmore C Bonte E Wall G Becker PB Dual regulation of the Drosophila hsp26 promoter *in vitro* Nucleic Acids Res 1995 23 2479 2487 10.1093/nar/23.13.2479 7630725 PMC307054

[B24] Wilkins RC Lis JT Dynamics of potentiation and activation: GAGA factor and its role in heat shock gene regulation Nucleic Acids Res 1997 25 3963 3968 10.1093/nar/25.20.3963 9321643 PMC147008

[B25] Akai J Kimura A Hata RI Transcriptional regulation of the human type I collagen alpha2 (COL1A2) gene by the combination of two dinucleotide repeats Gene 1999 239 65 73 10.1016/S0378-1119(99)00380-7 10571035

[B26] Ng TK Lam CY Lam DS Chiang SW Tam PO Wang DY Fan BJ Yam GH Fan DS Pang CP AC and AG dinucleotide repeats in the PAX6 P1 promoter are associated with high myopia Mol Vis 2009 15 2239 2248 19907666 PMC2774452

[B27] Htun H Dahlberg JE Topology and formation of triple-stranded H-DNA Science 1989 243 1571 1576 10.1126/science.2648571 2648571

[B28] Westin L Blomquist P Milligan JF Wrange O Triple helix DNA alters nucleosomal histone-DNA interactions and acts as a nucleosome barrier Nucleic Acids Res 1995 23 2184 2191 10.1093/nar/23.12.2184 7610046 PMC307006

[B29] Espinas ML Jimenez-Garcia E Martinez-Balbas A Azorin F Formation of triple-stranded DNA at d(GA.TC)n sequences prevents nucleosome assembly and is hindered by nucleosomes J Biol Chem 1996 271 31807 31812 10.1074/jbc.271.50.31807 8943221

[B30] Croston GE Kerrigan LA Lira LM Marshak DR Kadonaga JT Sequence-specific antirepression of histone H1-mediated inhibition of basal RNA polymerase II transcription Science 1991 251 643 649 10.1126/science.1899487 1899487

[B31] Lu Q Wallrath LL Granok H Elgin SC (CT)n (GA)n repeats and heat shock elements have distinct roles in chromatin structure and transcriptional activation of the Drosophila hsp26 gene Mol Cell Biol 1993 13 2802 2814 8474442 10.1128/mcb.13.5.2802-2814.1993 PMC359663

[B32] Strutt H Cavalli G Paro R Co-localization of Polycomb protein and GAGA factor on regulatory elements responsible for the maintenance of homeotic gene expression EMBO J 1997 16 3621 3632 10.1093/emboj/16.12.3621 9218803 PMC1169986

[B33] Lu Q Teare JM Granok H Swede MJ Xu J Elgin SC The capacity to form H-DNA cannot substitute for GAGA factor binding to a (CT)n*(GA)n regulatory site Nucleic Acids Res 2003 31 2483 2494 10.1093/nar/gkg369 12736297 PMC156050

[B34] Matharu NK Hussain T Sankaranarayanan R Mishra RK Vertebrate homologue of Drosophila GAGA factor J Mol Biol 400 434 447 10.1016/j.jmb.2010.05.010 20471984

[B35] Lehmann M Anything else but GAGA: a nonhistone protein complex reshapes chromatin structure Trends Genet 2004 20 15 22 10.1016/j.tig.2003.11.005 14698615

[B36] Barbeau B Bergeron D Beaulieu M Nadjem Z Rassart E Characterization of the human and mouse Fli-1 promoter regions Biochim Biophys Acta 1996 1307 220 232 8679708 10.1016/0167-4781(96)00060-7

[B37] Beaulieu M Barbeau B Rassart E Triplex-forming oligonucleotides with unexpected affinity for a nontargeted GA repeat sequence Antisense Nucleic Acid Drug Dev 1997 7 125 130 9149848 10.1089/oli.1.1997.7.125

[B38] Molano I Mathenia J Ruiz P Gilkeson GS Zhang XK Decreased expression of Fli-1 in bone marrow-derived haematopoietic cells significantly affects disease development in Murphy Roths Large/lymphoproliferation (MRL/lpr) mice Clin Exp Immunol 2010 160 275 282 10.1111/j.1365-2249.2009.04080.x 20015093 PMC2857951

[B39] Oates JC Levesque MC Hobbs MR Smith EG Molano ID Page GP Hill BS Weinberg JB Cooper GS Gilkeson GS Nitric oxide synthase 2 promoter polymorphisms and systemic lupus erythematosus in African-Americans J Rheumatol 2003 30 60 67 12508391

[B40] Martin-Donaire T Losada-Fernandez I Perez-Chacon G Rua-Figueroa I Erausquin C Naranjo-Hernandez A Rosado S Sanchez F Garcia-Saavedra A Citores MJ Vargas JA Perez-Aciego P Association of the microsatellite in the 3' untranslated region of the CD154 gene with rheumatoid arthritis in females from a Spanish cohort: a case-control study Arthritis Res Ther 2007 9 R89 10.1186/ar2288 17845713 PMC2212561

[B41] Wagener FA Toonen EJ Wigman L Fransen J Creemers MC Radstake TR Coenen MJ Barrera P van Riel PL Russel FG HMOX1 promoter polymorphism modulates the relationship between disease activity and joint damage in rheumatoid arthritis Arthritis Rheum 2008 58 3388 3393 10.1002/art.23970 18975324

[B42] Khani-Hanjani A Lacaille D Hoar D Chalmers A Horsman D Anderson M Balshaw R Keown PA Association between dinucleotide repeat in non-coding region of interferon-gamma gene and susceptibility to, and severity of, rheumatoid arthritis Lancet 2000 356 820 825 10.1016/S0140-6736(00)02657-X 11022930

[B43] Awad M Pravica V Perrey C El Gamel A Yonan N Sinnott PJ Hutchinson IV CA repeat allele polymorphism in the first intron of the human interferon-gamma gene is associated with lung allograft fibrosis Hum Immunol 1999 60 343 346 10.1016/S0198-8859(98)00133-5 10363726

[B44] Heubner M Wimberger P Kasimir-Bauer S Otterbach F Kimmig R Siffert W The AA genotype of a L1C G842A polymorphism is associated with an increased risk for ovarian cancer Anticancer Res 2009 29 3449 3452 19661372

[B45] Rebai M Kallel I Charfeddine S Hamza F Guermazi F Rebai A Association of polymorphisms in estrogen and thyroid hormone receptors with thyroid cancer risk J Recept Signal Transduct Res 2009 29 113 118 10.1080/10799890902845682 19519176

[B46] Takagi Y Masamune A Kume K Satoh A Kikuta K Watanabe T Satoh K Hirota M Shimosegawa T Microsatellite polymorphism in intron 2 of human Toll-like receptor 2 gene is associated with susceptibility to acute pancreatitis in Japan Hum Immunol 2009 70 200 204 10.1016/j.humimm.2009.01.006 19280717

[B47] Ye C Gao YT Wen W Breyer JP Shu XO Smith JR Zheng W Cai Q Association of mitochondrial DNA displacement loop (CA)n dinucleotide repeat polymorphism with breast cancer risk and survival among Chinese women Cancer Epidemiol Biomarkers Prev 2008 17 2117 2122 10.1158/1055-9965.EPI-07-2798 18708405 PMC2643086

[B48] Brouwer JR Willemsen R Oostra BA Microsatellite repeat instability and neurological disease Bioessays 2009 31 71 83 10.1002/bies.080122 19154005 PMC4321794

[B49] Asano Y Stawski L Hant F Highland K Silver R Szalai G Watson DK Trojanowska M Endothelial Fli1 deficiency impairs vascular homeostasis. a role in scleroderma vasculopathy Am J Pathol 2010 176 1983 1998 10.2353/ajpath.2010.090593 20228226 PMC2843486

